# Adolescent and Young Adult Preferences for the Delivery of Caring Contacts and the Incorporation of AI: Qualitative Interview Study

**DOI:** 10.2196/104853

**Published:** 2026-11-11

**Authors:** Kenneth Yi, Patrick Wedgeworth, Denise Chang, Sarah Danzo

**Affiliations:** 1 Department of Biomedical Informatics and Medical Education, University of Washington, Box 358047, Seattle, WA, 98195, United States, 1 847-398-4779; 2 Department of Psychiatry and Behavioral Sciences, University of Washington, Seattle, WA, United States; 3 Seattle Children's Hospital, Seattle, WA, United States

**Keywords:** Caring Contacts, suicide prevention, primary care, adolescents, young adults, mental health, depression

## Abstract

**Background:**

Caring Contacts is a low-cost and low-resource suicide prevention intervention that involves sending a series of nondemanding messages that convey care and support. While Caring Contacts is well studied among veteran and adult populations, intervention implementation preferences remain underexplored for adolescents and young adults, especially regarding its delivery in primary care settings. As primary care clinicians are often constrained in their capacity to respond to the time-sensitive needs of these patients while they wait to be connected to specialty care, identifying whether current technologies that can help alleviate these time barriers are acceptable to adolescents and young adult patients is an important next step.

**Objective:**

We evaluated the preferences of adolescents and young adults regarding the delivery of Caring Contacts. We also examined their perspectives on whether and how AI should be incorporated into the Caring Contacts intervention.

**Methods:**

Semistructured interviews were conducted with 10 adolescents and young adults recruited from outpatient health care clinics who reported a history of suicidal thoughts and behaviors. Participants were asked to reflect on their experiences and provide their perspectives on the intervention being delivered in a primary care setting. Interview transcripts were analyzed using qualitative coding techniques to evaluate their preferences and identify overarching themes.

**Results:**

Participants described a preference for receiving Caring Contacts via SMS text messaging, noting its perceived usability as the main contributor to this preference. While patient portals and physical letters were noted as other acceptable delivery methods, challenges to accessing them easily and in a timely manner made them less desirable compared with SMS text messaging. Regarding the incorporation of AI into Caring Contacts, participants generally viewed these technologies negatively and with hesitancy, describing them as potentially impersonal given the context of suicidality and wanting transparency if it were to be used.

**Conclusions:**

This study suggests that adolescents and young adults differ in their preferences for how they want Caring Contacts to be delivered compared with previously studied veteran and adult patient populations. AI may help address primary care clinician time and resource constraints, but its implementation must carefully address the concerns raised by adolescents and young adults, particularly around transparency in its use and its perceived impersonal nature.

## Introduction

Suicide is the second leading cause of death among adolescents and young adults (AYA) aged 10 to 34 years old in the United States [1]. Consequently, developing effective and accessible approaches to dynamically identify and respond to suicide risk that support AYA is imperative to improving AYA health outcomes. Digital health technologies (eg, mobile apps, chatbots, etc) offer the capabilities to address both timely and personalized aspects of care, but difficulties remain in relation to their implementation in practice [2].

Over the past decade, but particularly following the COVID-19 pandemic, there has been a significant rise in the use of telehealth and the prevalence of digital mental health interventions [3-5]. Recent developments in generative AI, such as large language models, have further contributed to this trend. While most applications of AI have been in the context of improving diagnostics, monitoring, and risk prediction models, applying AI to clinical interventions, such as Caring Contacts, remains underexplored [2,6].

Caring Contacts is a low-cost, low-resource, evidence-based intervention that involves sending caring, nondemanding, supportive messages to patients with suicidality [7]. Although the Caring Contacts intervention was initially delivered through physical mailed letters, more recent studies have begun to examine the implementation of Caring Contacts via text messaging approaches, as well [7,8]. However, many of these studies examine Caring Contacts in a single form (eg, caring letters and caring texts), resulting in limited exploration of recipients’ preferred delivery methods. Moreover, much of the current literature on Caring Contacts has focused on the acceptability and effectiveness of the intervention in adult populations (eg, military personnel and veterans). Studies evaluating Caring Contacts with AYA remain sparse and largely focus on AYA in high-acuity settings (eg, psychiatric inpatient care and emergency department discharge) [7,9-12]. Thus, more research is needed to evaluate Caring Contacts implementation with AYA, particularly in lower-acuity settings such as primary care.

Emerging research suggests that AYA who receive Caring Contacts through text message prefer the option to respond (rather than receiving a text without the ability to respond) [13]. However, enabling 2-way messaging increases the burden placed upon clinicians, particularly those in busy primary care settings. This can reduce clinician buy-in and the sustainability of Caring Contacts implementation. AI-supported systems, such as automated priority classification of messages and adaptive message templates, could help facilitate 2-way Caring Contacts while mitigating clinician time constraints. However, how AI-based systems in this specific health care context are perceived by AYA is unknown.

Primary care represents a particularly promising area for this intervention as it is a frequently accessed health care setting among AYA and their families [14,15]. While increased screening for suicidal thoughts and behaviors (STB) in primary care has led to better detection of STB, primary care providers (PCPs) face several barriers to managing risk once it is detected [16,17]. Additionally, PCPs routinely refer AYA with STB to specialty mental health care. However, patients often face long wait times accessing these services [18,19]. This results in PCPs being tasked with managing STB until patients are connected. Unfortunately, PCPs are often constrained in their capacity to manage STB due to limitations in time, resources, and training [20-24]. Digitally delivering Caring Contacts may offer a practical solution to these constraints while extending support to AYA between visits and during their wait to be connected to specialty care.

Given the heightened risk for suicide during adolescence and young adulthood, an important first step before further developing these systems is to understand how AYA expressing STB would prefer to receive Caring Contacts in outpatient primary care contexts and if AI involvement is appropriate. Incorporating these preferences for Caring Contacts and AYA feedback for adapting the intervention to better meet their needs is integral to effective implementation and sustained engagement [5,25]. The goal of this exploratory study was to elicit and better understand AYA preferences for the delivery of Caring Contacts. We were also interested in exploring AYA’s perspectives on the potential incorporation of AI into Caring Contacts as an emerging consideration in digital mental health.

## Methods

### Ethical Considerations

All study procedures were approved by the University of Washington Institutional Review Board (IRB) under the study identification number STUDY00020677 (approved July 2024). The University of Washington and its IRBs follow recommendations for participant safety given by the Belmont Report and Declaration of Helsinki. Assent, consent, and parental permission forms were provided to all participants. A trained research staff member met with potential participants to verbally review consent documents. After reviewing the document together, participants were given an opportunity to ask questions and read through the document on their own. Research staff answered any questions and confirmed participant understanding prior to obtaining consent. Research staff conducting consent documented informed assent, consent, or parental permission, including the date of assent, consent, or parental permission; the research team member conducting consent; and confirmation that (1) the assent, consent, or parental permission document was provided to the participant; (2) the participant reported understanding of the study; (3) the participant’s questions were answered; and (4) the participant provided verbal assent, consent, or parental permission to participate in the study. Documentation for youth (aged <18 years) who assented to participate also included the date that the parental permission form was provided to parents and documentation of whether the parent provided verbal permission for their child to participate. If parental permission was given, the date on which parental permission was obtained and the research staff member who reviewed the parent permission form with the parent were also documented. Information regarding the voluntary nature of the study, information about confidentiality and privacy, and information regarding how to withdraw from the study were provided as part of informed assent, consent, or parental permission. Each participant was compensated with a US $50 gift card for taking part in the study.

### Participants and Study Design

This study drew on a larger project that examined in-depth perspectives of both AYA and caregivers regarding suicide prevention interventions. Participants were asked to reflect on their experiences and perceptions of the interventions being delivered in a primary care setting. As AYA are the intended recipients of Caring Contacts, this paper focuses specifically on their preferences regarding delivery method and the incorporation of AI. Caregiver findings and AYA preferences not related to AI and the delivery method of Caring Contacts (eg, content, frequency of messages, and duration of the intervention) were also examined in the larger project but are beyond the scope of this specific study. Participant identification numbers were assigned sequentially across all AYA and caregivers. As a result, the identification numbers of the AYA participants are not consecutive and do not reflect the total number of AYA that participated in this study.

Participants were recruited from outpatient health care clinics (including primary care, outpatient medical clinics, and outpatient mental health clinics) in Washington state. Recruitment occurred via email advertisements, flyers, and clinician referrals.

A target sample of 10 AYA participants was set a priori, consistent with the exploratory aims of this formative study and partly because of resource constraints. The goal was to gather preliminary, in-depth information on AYA receptiveness to and perceptions of suicide prevention interventions to inform subsequent development. Given the narrow aim of the study and specificity of the sample, this sample size was determined to be sufficient for the purposes of this study [26]. Recruitment to the point of thematic saturation was therefore not a primary design goal.

Participants consisted of AYA aged 13 to 30 years old (n=10). Participant eligibility included the following inclusion criteria: English-speaking AYA aged 13 to 30 years with a history of self-reported STB [27].

To determine if participants met eligibility for having a history of STB, participants responded to 3 items on the study interest survey: (1) Have you or someone you care about experienced suicidal thoughts or feelings in the past? (2) Have you or someone you care about ever received any type of mental health intervention or treatment? and (3) Please tell us why you are interested in participating in this study. Individuals who answered “no” to the first 2 items and did not indicate a history of STB in the free response item were deemed ineligible for the study. Individuals who answered “yes” to either of the first 2 items or provided a background of STB in the free response box were invited to meet with a study staff member who verbally assessed if the individual had current or past STB.

No participants who reviewed the informed consent form declined to participate in the study. However, 4 adolescents and 6 young adults who expressed interest in the study via the study interest survey were unable to be reached to schedule a time to review consent. Additionally, 2 AYA were excluded because they denied a history of STB, and 1 was excluded because they expressed interest after the study recruitment period had closed. A participant flowchart summarizing our recruitment process can be found in Multimedia Appendix 1.

### Data Collection Methods

Semistructured interviews were conducted by 2 research team members (SD and DC) over Zoom (Zoom Communications, Inc). Interviewers included a clinical psychologist (PhD) and a psychiatrist (MD) with backgrounds in primary care behavioral health and adolescent and young adult mental health. Interviewers had extensive clinical interviewing experience and completed several practice interviews prior to study data collection to ensure standardization across interviewers. Interviewers had no relationship with participants prior to study enrollment. Individual interviews lasted approximately 60 to 90 minutes and asked participants for their perspectives regarding Caring Contacts and feedback on how the intervention should be delivered in primary care settings. During the interview, interviewers introduced themselves as researchers but did not disclose other personal information. The motivation behind the research had been explained to participants in the consent form. All participants were introduced to the concept of Caring Contacts with a brief prompt explaining how some people find it helpful to receive supportive messages from their care team when they are experiencing suicidal thoughts and feelings. Example messages from a program at a large children’s hospital were shown when participants had difficulty thinking of supportive messages they would find helpful or when they wanted additional clarification about the kinds of messages they might receive. The complete AYA interview guide can be found in Multimedia Appendix 2. Of note, this paper draws specifically from only the first part of the interview, which focuses on Caring Contacts.

Participants also completed a brief self-report questionnaire that asked about demographic information including age, race, ethnicity, and sex.

### Data Analysis

Interviews were audio recorded and transcribed. Interview transcripts were cleaned and then analyzed using ATLAS.ti (version 25.0.1; Lumivero), software designed for qualitative coding and analysis [28].

Data were analyzed using a codebook approach to thematic analysis, which combines the structured procedures of coding reliability thematic analysis with the interpretive orientation of reflexive thematic analysis [29]. We selected this approach because it was flexible enough for our formative aims and best accommodated both structural codes reflecting the interview guide’s question structure and inductively derived thematic codes. It also helped ensure consistency across coders.

For the first round of coding, 2 coders (PW and KY) independently familiarized themselves with an initial set of 6 transcripts, encompassing both AYA and caregivers, and inductively generated an initial codebook for both structural (ie, based on specific questions asked) and thematic (ie, recurring themes) codes. Coders consisted of a physician (MD) and a graduate student (MS), both of whom had prior training and experience in qualitative coding. Following the first round of coding, coders met weekly to determine qualitative code definitions and to resolve any discrepancies. The 2 coders then individually examined 2 randomly selected transcripts from the initial set. After this round of coding, overlapping thematic codes were discussed and merged for clarity. Coders then revisited the same transcripts for another round of coding. Following this, intercoder agreement was assessed and indicated satisfactory reliability as a Krippendorff cu-α of 0.89 for structural codes and 0.80 for thematic codes was achieved [30,31]. The remaining transcripts were coded independently. Throughout this process, coders flagged any content not captured by the existing codebook, which was discussed at weekly meetings and accommodated through refinements to existing code definitions. No new thematic codes were identified or added after the initial set of 6 transcripts. Although recruitment was not designed around saturation, this suggests codebook stability and code saturation–the point at which researchers have “heard it all,” though not necessarily meaning saturation, the point at which they “understand it all” [32]. After all transcripts were coded, PW and KY met again to finalize code definitions. The codebook can be found in Multimedia Appendix 3.

## Results

### Overview

The participants that completed individual interviews consisted of 10 AYA with a mean age of 22.50 (SD 5.08) years. Among the 10 participants, 2 were 18 years old or younger and were classified as adolescents (mean 15.0, SD 2.83). Eight participants were between age 18 and 30 years and were classified as young adults (mean 24.38, SD 3.46). Participant demographics are reported in Table 1.

**Table 1.** Participant demographic characteristics (n=10).

Characteristics	Participants
Age (y), mean (SD; range)	
Adolescents and young adults (13-30 y)	22.50 (5.08; 13-28)
Adolescents (≤18 y)	15.00 (2.83; 13-17)
Young adults (≥18 y)	24.38 (3.46; 20-28)
Sex, n (%)	
Male	5 (50)
Female	4 (40)
Other or prefer not to say	1 (10)
Race (n=11)^a^, n (%)	
Asian	1 (9.1)
Black or African American	4 (36.4)
White	6 (54.5)
Ethnicity, n (%)	
Hispanic	2 (20)
Non-Hispanic	8 (80)

a Participants could select all applicable racial categories; therefore, the total may exceed the number of participants.

Participant responses were open-ended and categorized based on structural and co-occurring thematic codes. Participants’ support for or opposition to each delivery method was determined based on these categorizations and was coded only when explicitly mentioned. Descriptions of preference reflect the number of participants who expressed support for or opposition to each delivery method. Participants were not asked to formally rank their preferences as part of this study, and no additional probes were used to elicit comparisons between delivery methods. Because the study included only 2 adolescent participants and the themes reported below were present across age groups, results are reported at the level of AYA rather than by subgroup. An overview of this, along with corresponding quotations, can be found in Multimedia Appendix 4. In the following section, we present findings from both (1) AYA preferred delivery methods and (2) AYA’s response to AI for Caring Contacts. Additionally, we provide an overview of the structural code responses and a more in-depth discussion of the thematic analysis.

### Preferred Delivery Methods for Caring Contacts

To better understand AYA’s preferred delivery methods for Caring Contacts, each participant was asked, “How would you want to receive these messages?*”* Most participants (n=8) indicated “SMS text messaging” as their most preferred delivery method for receiving Caring Contacts. No participants reported not wanting to receive Caring Contacts messages through SMS text messaging. Although several participants also noted that “patient portal” (n=5) and “letter” (n=4) would be acceptable methods, they each had a nearly equal number of AYA indicating they would not prefer these methods (“patient portal”: n=3; “letter”: n=3). “Email” received modest support (n=3) with no opposition from any participant. Compared with these more traditional delivery methods, “apps” (n=1) and “audio messages” (n=1) were identified as having desirable characteristics for the purposes of Caring Contacts. Although these 2 modalities represent potentially promising and novel areas to investigate further, our participants did not discuss them in-depth. Overall, responses in this section tended to relate to the ease of use for each individual delivery method.

### Perceived Usability of Delivery Methods

Participants identified multiple reasons why certain delivery methods were preferable, with a common sentiment being the method’s perceived usability. One participant summarized this aspect, stating:

*I would prefer a text message just because it’s the quickest way to reach me.*

[P4]

In addition to its ubiquity, participants noted how the asynchronous nature of SMS text messaging affords a reduced pressure to respond compared with modalities such as phone calls.

AYA participants had mixed perspectives on the use of patient portals such as Epic’s MyChart. Those in support of the delivery method ranked it as secondary to SMS text messaging. Those who did not prefer patient portals identified difficulty navigating its different features and receiving notifications in a timely manner as the main challenges to its use. One participant also expressed concerns about the connotations associated with information accessed via patient portals:

*I think staying away from too serious of a spot, like a patient portal. That’s where I go to tell someone I think I’m going to die and I pay bills. You know, I wouldn’t go looking there for a nice message.*

[P1]

Similar to patient portals, participants were also split on Caring Contacts being delivered via letters in the mail. While there was some enthusiasm for their novelty within a predominantly digital era and as a welcome surprise to occasionally receive, many expressed concerns regarding their use in practice, especially as a primary delivery method for Caring Contacts. Speaking on receiving Caring Contacts in the mail, one participant commented:

*I’d say I lean more towards digital just because I don’t frequently check my mail. So if you send me something in my mail, I might not see it for a very long time.*

[P5]

One participant brought up concerns around privacy, stating:

*And if it’s like via physical mail then lack of privacy. Because like parents or something might open it.*

[P14]

One participant also discussed logistical concerns around changing addresses:

*And also with letters, like I love the letters idea. It’s so cute, but you need a consistent address. And especially if you’re sending them out over long periods of time, usually if you’re renting, you got to move...*

[P1]

Unlike the delivery methods listed above, about which participants expressed strong opinions, responses that pertained to the use of email had a more neutral tone associated with them. Email was often viewed as less desirable in relation to both SMS text messaging and patient portals. One participant stated:

*Email is okay, but not as preferable just because I feel like it’s easy to get lost in the inbox and whatnot.*

[P10]

### Response to AI for Caring Contacts

#### Overview

After being introduced to Caring Contacts and discussing preferences about its delivery, each participant was asked, “How would you feel if the care messages were generated by an AI-driven template (informed by your clinical information but still reviewed and approved by your care team/provider)?” While a few AYA indicated a more neutral or positive stance on this topic, most participants responded negatively to the possibility of incorporating AI into Caring Contacts. In general, concerns revolved around 2 main aspects: the impersonal nature of this method and the potential lack of transparency in its use.

#### Impersonal Nature of AI

The most significant concern about the implementation of AI in Caring Contacts expressed by participants was the potential for the intervention to feel isolating and impersonal. Delegating its delivery, and especially its content, to an AI tool was perceived as insensitive to the personal nature of mental health and, more specifically, suicidality. Reflecting upon how they would respond to the use of AI for Caring Contacts, one participant stated:

*Honestly, I probably wouldn’t feel great about it. I had a teacher write me like a school recommendation using AI and it’s like, wow, it hurt a little bit. I get that it’s a lot easier, but it feels so like impersonal, I guess. Especially if it’s a doctor you’ve been seeing like throughout your life where they get to know you and they know a lot about you and it’s like I think it feels more personal for them to be the one reaching out.*

[P2]

Unlike individuals from their care team, with whom patients can develop connections and rapport over time, AI may lack a personal connection. One participant expressed this concern:

*I don’t think [AI] would be as effective. This might just be personal bias, but I’m not really a fan of interacting with AI. It feels very fake, especially for something that’s as personal as mental health.*

[P14]

#### Transparency in the Use of AI

In line with participants expressing concern around the potential impersonal nature of automating aspects of Caring Contacts by incorporating AI, several participants also discussed the importance of transparency in the use of AI systems when they are incorporated in mental health interventions. For example, one participant recommended:

*I think you should just be very clear like when it is or is not an AI, especially if it’s happening through text. Because I think people, when they feel like they’re being deceived or tricked or like when someone’s trying to deceive or trick them through AI, it sucks worse because it’s like someone put up like a cutout instead of having a real human being talk to you. And that’s like...how much more alone can you feel in a moment you know like even the person that’s supposed to be there isn’t really there.*

[P1]

One participant also expressed concern, noting:

*I feel like if it was just—like currently if I answer on a survey that I have suicidal or self-harming thoughts, like I get an automatic reply about like ‘call this number’ or things like that. Which doesn’t really seem helpful to me. Because it feels like automated...*

[P14]

Participants in support of the incorporation of AI into Caring Contacts pointed to the improved response times and availability as positives. However, they also highlighted several qualifications for that support. For example, many participants stated that these methods would need to be approved by their clinicians to be more acceptable. One participant noted:

*If it’s approved by my clinician, that would be great. If I’m able to sense that I’m getting a robot text, I don’t think I would like that.*

[P4]

Most participants expressed skepticism and hesitancy around the use of AI in care for their mental health. One participant summarized this sentiment:

*I’m glad you guys are utilizing tools. Which is great. You know, AI is the future however it’s just very far removed from like, I just feel like if you’re going to work in medicine you have to be more personable. You have to be more intentional. And you have to put more care into your work.*

[P11]

## Discussion

### Principal Findings

The goal of this study was to better understand AYA’s perspectives on how Caring Contacts should be delivered in primary care settings and their perspectives on incorporating AI into Caring Contacts. Overall, results suggest that AYA viewed the perceived ease of accessing and engaging with Caring Contacts messages as being the most important factor to consider for the delivery of these supportive messages.

As text-based messaging platforms are a predominant mode of communication and interaction among AYA, it comes as little surprise that participants generally preferred SMS text messaging and often pointed to its ease of use and familiarity as a major contributing factor for this preference [33,34]. Participants noted how the asynchronous nature of SMS text messaging affords reduced pressure to respond, which matches the nondemanding intent of Caring Contacts. AYA preferences for SMS text messaging also align with findings from Kornfield et al [35], who reported high levels of user engagement and positive feedback among young adults (aged 18‐25 years) participating in a mental health text messaging intervention.

As noted, there has been limited exploration of recipients’ preferred delivery methods in this literature. Among studies that have elicited these preferences, Ammerman and colleagues [36] found that veterans aged 50 years or older showed a greater preference for physical letters as their primary method for receiving Caring Contacts. Similarly, Reger and colleagues [37] found that high-risk veteran psychiatric inpatients most preferred receiving Caring Contacts in the form of a letter or postcard, as well, while text message was their least preferred method.

In contrast, the current study adds to the growing literature that AYA prefer digital delivery methods (ie, SMS text messaging) for Caring Contacts rather than physical methods (ie, letter) due to their comfort around these technologies and the increased perceived privacy and security these methods afford. AYA preferences for SMS text messaging also align with recent work examining Caring Contacts in the context of transgender and gender-diverse adolescents receiving care from a specialty gender clinic, Black adolescents being seen in an emergency department for STB, and high-risk adolescents after discharge from acute mental health services [38-41]. These findings together suggest that AYA generally lean more toward digital delivery methods for Caring Contacts. However, the success of these interventions hinges upon having access to a device and ensuring the privacy of its content. With AYA populations, but particularly adolescents, a notable barrier is how to navigate situations where a parental figure might screen the messages they receive or revoke their access to these devices.

While AYA generally felt positive about receiving supportive messages via text, there was more skepticism and hesitancy around the incorporation of AI, especially if it was used to automatically respond to patient messages. Participants expressed the importance of having a relationship with the sender and being able to sense the genuine intent behind the message for it to feel supportive. Whether AI-based systems can replicate and convey these qualities remains an open question. Although participants recognized that automated methods could generate a faster response, concerns that these supportive messages would feel impersonal outweighed this potential benefit. Thus, with the increasing prevalence of generative AI and the automation of tasks, developers of digital health tools for Caring Contacts and mental health overall need to be particularly careful not to further isolate individuals experiencing STB.

### Limitations and Future Directions

This study has several limitations, with the most notable being our limited sample size. Because the aims of the study were exploratory in nature and meant to elicit initial feedback for future work, data collection did not continue to the point of thematic saturation for AYA separately. As a result, the preferences described here should not be assumed to be exhaustive, and additional perspectives likely exist that were not represented in these interviews. The current study provides important preliminary data; however, future research should examine these questions with a larger participant sample to determine if these initial findings extend to a larger AYA patient population. Additionally, our study only included English-speaking AYA by design, due to funding and resource limitations. An important consideration for future directions would be to recruit AYA with a preference for languages other than English as well as participants encompassing a wider range of races and ethnicities.

Furthermore, while we did not anticipate or observe meaningful differences between young adults and younger adolescents, future studies could test if differences exist at this more granular level. For example, digital, health, and overall literacy exist on a continuum that can differ meaningfully between AYA. Moreover, not all adolescents may have access to a personal digital device, and those who do may not be able to use it privately. All of these factors can influence participants’ responses regarding preferred delivery methods and views around the use of AI and therefore should be further explored in future research.

While patient portals can offer increased security, privacy, and ease of implementation for health care systems, both AYA in our study expressed difficulty using them, which contributed to their preferences for SMS text messaging. Future work could also examine AYA proficiency in navigating digital health infrastructure and their potential needs in this space. Since our participants considered letters strictly in their physical form, another potential delivery method to be further explored for Caring Contacts could be digital letters and postcards. These methods can support not only more traditional text and image messages but also audio and video functionalities.

Preferences around delivery method are also shaped by other message-related elements, including the sender, content, length, timing, and cultural appropriateness. This analysis examined delivery method in isolation from these components. Recent qualitative work by Vidal and colleagues [39] explored adolescent perspectives on these elements following emergency department care, yet a gap remains for primary care and outpatient medical settings. Examining how these elements interact with delivery preferences in lower-acuity settings would provide a more complete account of how this intervention should be adapted for AYA.

This study also relied on open-ended questions to examine AYA preferences. As participants were not asked to explicitly rank delivery methods and their responses were categorized during coding, the relative ordering of preferred delivery methods should be interpreted with caution. Similarly, given the ubiquity of AI and its large scope of applications and capabilities, we acknowledge that our description of AI still leaves considerable room for subjective interpretation based on past personal experiences. Future work could use structured rankings to confirm delivery preferences. Studies could also examine more specific nuances and logistics around what AI involvement could entail and how both AYA view the technology.

Despite these limitations, our study represents one of the first to examine AYA perspectives on the delivery of the Caring Contacts intervention in a primary care setting, as previous studies have focused more on its effectiveness and implementation in other settings [24,41,42]. Although more work needs to be done to confirm the generalizability of our findings, our initial results suggest that AYA have specific preferences regarding the delivery of Caring Contacts that differ from those of previously studied adult veteran populations and warrant consideration to inform implementation.

### Conclusions

Because most of the existing research on Caring Contacts focuses on high-risk adult populations and limits evaluation to a single delivery method, it is critical that future work continues to intentionally incorporate the perspectives of AYA to better adapt this intervention for this population [9-11]. Our findings suggest that AYA preferences for Caring Contacts delivery differ in important respects from those documented among veteran and older adult populations but remain similar in the sentiment that these messages need to be personal and genuine for recipients to feel cared for [36,37,43]. These findings will inform the next phase of this work, in which a Caring Contacts prototype reflecting AYA-identified preferences will be evaluated for usability, feasibility, and acceptability. Accounting for AYA preferences is especially important given the concerning rise in mental health symptoms and suicide-related deaths among AYA in recent years [44]. In high-stakes clinical contexts, such as with AYA expressing STB, there exists a tension between timely support, the bandwidth of primary care clinicians, and current technologies that can afford that immediacy. As digital mental health interventions continue to evolve, identifying safe, acceptable, and effective methods for delivering Caring Contacts and understanding if and how AI can be integrated will be essential for ensuring that clinical teams can address the urgent mental health needs of AYA.

## Supplementary material

10.2196/104853 Multimedia Appendix 1 Flow diagram of recruitment, enrollment, participation, and analysis.

10.2196/104853 Multimedia Appendix 2 Semistructured interview guide used for adolescents and young adults.

10.2196/104853 Multimedia Appendix 3 Final version of qualitative codebook containing both thematic and structural codes.

10.2196/104853 Multimedia Appendix 4 Table summary of qualitative themes with their related question and corresponding quotations.

## Abbreviations

AYA: adolescents and young adults
IRB: Institutional Review Board
PCP: primary care provider
STB: suicidal thoughts and behaviors
