# Supplementary material for: Adolescent and Young Adult Preferences for the Delivery of Caring Contacts and the Incorporation of AI: Qualitative Interview Study
Source: JMIR Form Res. 2026 Nov 11;10:e104853. doi: 10.2196/104853 (PMC13754059; doi:10.2196/104853)
Supplement: Supplementary file 1 [file formative-v10-e104853-s001.docx]

**Multimedia Appendix 1. CONSORT Diagram**

Flow diagram of recruitment, enrollment, participation, and analysis

Analyzed (n=10)

Excluded from analysis (n=0)

Analysis

**Assessed for eligibility (n= 23)**

**Completed Interview (n=10)**

Excluded (n=13)

Not meeting inclusion criteria (n=2)

Declined to participate (n=10)

Study enrollment had closed (n=1)

**Enrolled (n=10)**
