# Supplementary material for: Adolescent and Young Adult Preferences for the Delivery of Caring Contacts and the Incorporation of AI: Qualitative Interview Study
Source: JMIR Form Res. 2026 Nov 11;10:e104853. doi: 10.2196/104853 (PMC13754059; doi:10.2196/104853)
Supplement: Supplementary file 2 [file formative-v10-e104853-s002.docx]

***Multimedia Appendix***

Multimedia Appendix 2. Interview Guide

**AYA Interview Guide**

*Interview is semi-structured so interviewer can ask any follow-up questions that come to mind.*

1. **When people are feeling down and having suicidal thoughts, some people find it helpful to receive supportive messages from their care team. If you were experiencing these kinds of thoughts and feelings, would you want to receive supportive messages from your care team?**

**If you were to receive supportive messages from your care team….**

a. **Who from the care team would you want to receive these messages from? (**e.g., from a PCP, other primary care staff, a counselor, someone not involved in your care, the clinic as a whole, etc.)
b. **What kind of messages would you find helpful or supportive? Can you think of some examples?** (examples of supportive messages provided)
   i. “Wherever you are today, you are loved, valued, and strong”
   ii. “Accepting support is a sign of strength”
   iii. “It’s ok to take a break”
   iv. “Your emotions are valid, and you are enough”
   v. “It’s ok to be kind to yourself”
   vi. “You are not alone”
   vii. “Mistakes are normal and they are not the whole story”
   viii. “You are becoming who you were meant to be”
c. **How would you want to receive these messages?** For example, would you want a letter in the mail, a text message, an email, or a message through a patient portal or an app?
d. **How often do you think these messages should be sent?** *(x times a week or month)*
e. **How long do you think they should be sent for? (***x number of weeks/months; x number of messages total)*
f. **Would you like to receive messages in multiple ways or have one consistent communication method?** (for ex, sometimes a mailed letter and other times a text; or – always a text)
g. **Do you think these messages should just be caring messages or should they include any additional content (**such as information about crisis lines, coping skills, or other educational materials to help manage suicidal thoughts**)?**
h. **How would you feel if the care messages were generated by an AI driven template** (informed by your clinical information but still reviewed and approved by your care team/provider)**?**

2. **Would it be helpful for the care team to contact you to check-in about how you are feeling and ask you about the suicidal thoughts?**
   a. **If yes, should this happen along with the caring message or at a separate time?**

**If the care team were to reach out to you to check-in about how you are doing….**

b. **How would you want to be contacted?** (ex., call, text, email, Mychart, via an app, other)?
c. **How would you want to be asked about how you are doing**? (e.g., open ended response, options to choose from [multiple choice], fill out a survey or questionnaire, something else?
d. **How would you want your messages to be tailored?** (for example, take into account what you said two weeks ago into the check-in message)
e. **How frequently would you want to be asked about how you are doing**? (e.g., once a week/month, etc.)
f. **How long do you think check-ins should happen? (***x number of weeks/months; x number of messages total)*
g. **Would you want a response if you indicate that you are not doing well or are having worsening suicidal thoughts**?
   i. **If yes, what kind of response/message do you think would be helpful?**
h. **How would you want someone to respond**? (ex., call, text, email, Mychart, via an app, other)?
i. **How would you feel if the check-ins were done via AI or a chatbot, instead of a human?**
j. **Patient responses are confidential, with the exception of situations where there is serious concerns about a patient’s safety. Outside of these exceptions, would you want your responses/information to be shared with anyone (e.g., your parents/caregiver, clinical provider, etc.)?**
   i. **If yes, *who* would you want this information shared with and *what* information would you want to be shared?**

3. **General questions:**
   a. **Do you have any concerns about accessibility (physical and technical capabilities of being able to receive supportive messages or being contacted for check-ins)?**
   b. **What barriers might get in the way of receiving messages or being able to interact with the care team** (e.g., being grounded and having phone taken away; not going into the patient portal because of dual identity verification, etc.)**?**

c. **Do you have any concerns about privacy or security with receiving or responding to messages from your care team?**
