# Supplementary material for: Adolescent and Young Adult Preferences for the Delivery of Caring Contacts and the Incorporation of AI: Qualitative Interview Study
Source: JMIR Form Res. 2026 Nov 11;10:e104853. doi: 10.2196/104853 (PMC13754059; doi:10.2196/104853)
Supplement: Supplementary file 3 [file formative-v10-e104853-s003.docx]

***Multimedia Appendix***

Multimedia Appendix 3. Final Codebook

**Codebook**

| **Thematic Codes** | |
| --- | --- |
| *Autonomy* | |
| Concern for harm from institution interventions | Patient’s concern for harm from institutional interventions such as involuntary hospitalization or police encounters |
| Patient Choice | Design the intervention in a way that is not rigid and preserves patient choice |
| Transparency | Components of the intervention should be designed in a clear and transparent manner |
| *Communication Appropriateness* | |
| Patient-directed communication style | Use communication styles that take into account the patient's age and context |
| Provider authenticity | Maintain authenticity to who you are when communicating with the patient |
| Timely response | Make sure intervention/response from care team happens in a timely manner |
| Tone appropriateness | The provider's tone needs to be appropriate to the situation |
| Up-to-date STB history | Although suicidality was mentioned in the past, this does not mean the patient is in the same headspace currently |
| Appropriate word choice | The provider's choice of words needs to be appropriate for the context and situation |
| - Age | Use communication styles that take into account the patient's age |
| - Avoid patronizing language | Avoid language that looks down on or patronizes the patient |
| - Avoid platitudes | Avoid language that is generic and feel-good |
| - Avoid victim blaming | Avoid blaming the patient for their own suicidality |
| - Health literacy | Use communication styles that take into account the patient's health literacy |
| *Emotional Connection and Support* | |
| Importance of boundaries | Use where the participant is more comfortable when the intervention is non-judgmental and professionally distant, as opposed to close and friendly |
| Isolation/Alienation/Impersonal | This should be used for things that are isolating/alienating or impersonal to the patient |
| Provider support | Use for areas where providers specifically could provide more support to patients |
| Therapeutic relationship | Use where the idea of a long-term therapeutic relationship between provider and patient is considered beneficial |
| Provider affirmation | Affirmations of the patient's worth, experiences, feelings, efforts, etc. |
| - Belonging | Discussion that references the benefit of making a patient feel like they belong or are cared about by others in their life |
| - Encouragement | Use for areas where encouraging communication is discussed or requested |
| *Systemic Factors* | |
| Beneficence | Do not violate what is good for the patient, even if the patient requests it |
| Learned helplessness | Avoid promoting learned helplessness in patients |
| Liability | This should be used whenever there is concern an approach would lead to legal liability |
| Risk of harm | This should be used whenever there is concern an approach would lead to bad outcomes or errors |
| Mental health competency | Discussions around needing expertise or competency around suicidality and mental health |
| Stigma toward suicidality and/or treatment | Discussions around cultural views of suicide that need to be considered |
| Usability | This should be used whenever how easy or difficult something to use or navigate is brought up |
| *Treatment Personalization* | |
| Acuity dependent | Design intervention that changes options based on patient acuity |
| Kind gestures | Can include things like a coffee gift card, snacks, etc. |
| Self-care skills | Skills that promote self-care approaches to suicidality |
| **Structural Codes** | |
| *Part 1* | |
| AI/Automated CC | Unsure |
|  | Negative |
|  | Neutral |
|  | Positive |
|  | NOS* |
| CC Expectation | One-way |
|  | Two-way |
|  | Multi-way |
|  | Unsure |
|  | NOS* |
| CC Content | Educational content = No |
|  | Educational content = Yes |
|  | Helpline = No |
|  | Helpline = Yes |
|  | No additional content |
|  | Unsure |
|  | NOS* |
|  | Other/Unspecified additional content |
| CC Duration | For a month or less |
|  | For a week or less |
|  | For a year or less |
|  | More than a year |
|  | NOS* |
|  | Unsure |
| CC Kind of Message | NOS* |
| CC Response | Expected |
|  | Unsure |
|  | Optional |
|  | NOS* |
| CC Sender | Unsure |
|  | NOS* |
|  | Other individual |
|  | Case manager/Social worker = No |
|  | Case manager/Social worker = Yes |
|  | Non-primary care provider = No |
|  | Non-primary care provider = Yes |
|  | Mental health professional = No |
|  | Mental health professional = Yes |
|  | Primary care provider = No |
|  | Primary care provider = Yes |
| CC Delivery Method | App = No |
|  | App = Yes |
|  | Audio text = No |
|  | Audio text = Yes |
|  | Email = No |
|  | Email = Yes |
|  | Letter = No |
|  | Letter = Yes |
|  | Patient portal = No |
|  | Patient portal = Yes |
|  | Phone = No |
|  | Phone = Yes |
|  | Social media = No |
|  | Social media = Yes |
|  | Text = No |
|  | Text = Yes |
|  | NOS* |
|  | Unsure |
| CC Modality | Single Modality |
|  | Multiple Modalities |
|  | NOS* |
|  | Unsure |
| CC Sent | More than daily |
|  | Daily or less |
|  | Weekly or less |
|  | Monthly or less |
|  | NOS* |
|  | Unsure |
| Receive Caring Contacts | No |
|  | Yes |
|  | NOS* |
|  | Unsure |
| *Part 2* | |
| AI/Automated Check-ins | Unsure |
|  | Negative |
|  | Neutral |
|  | Positive |
|  | NOS* |
| Check-in Delivery Method | App = No |
|  | App = Yes |
|  | Audio text = No |
|  | Audio text = Yes |
|  | Email = No |
|  | Email = Yes |
|  | Letter = No |
|  | Letter = Yes |
|  | Patient portal = No |
|  | Patient portal = Yes |
|  | Phone = No |
|  | Phone = Yes |
|  | Social media = No |
|  | Social media = Yes |
|  | Text = No |
|  | Text = Yes |
|  | NOS* |
|  | Unsure |
| Check-in Duration | For a month or less |
|  | For a week or less |
|  | For a year or less |
|  | More than a year |
|  | NOS* |
|  | Unsure |
| Check-in Modality | Single Modality |
|  | Multiple Modalities |
|  | NOS* |
|  | Unsure |
| Check-in Response | Expected |
|  | Optional |
|  | NOS* |
|  | Unsure |
| Check-in Structure | Open-ended |
|  | Structured |
|  | Both |
|  | NOS* |
|  | Unsure |
| Check-in Tailored | No |
|  | Yes |
|  | NOS* |
|  | Unsure |
| Check-in Kind of Message | NOS* |
| Check-in Sent | More than daily |
|  | Daily or less |
|  | Weekly or less |
|  | Monthly or less |
|  | NOS* |
|  | Unsure |
| CC+Check-ins | Both |
|  | Combined |
|  | NOS* |
|  | Separate |
|  | Unsure |
| Receive Check-ins | No |
|  | Yes |
|  | NOS* |
|  | Unsure |
| *Part 3* | |
| Receive STB Response | Unsure |
|  | No |
|  | Maybe |
|  | Yes |
|  | NOS* |
| STB Response | App = No |
|  | App = Yes |
|  | Audio text = No |
|  | Audio text = Yes |
|  | Email = No |
|  | Email = Yes |
|  | Helpline = No |
|  | Helpline = Yes |
|  | In-person = No |
|  | In-person = Yes |
|  | Patient portal = No |
|  | Patient portal = Yes |
|  | Phone = No |
|  | Phone = Yes |
|  | Resources = No |
|  | Resources = Yes |
|  | Schedule appointment = No |
|  | Schedule appointment = Yes |
|  | Text = No |
|  | Text = Yes |
|  | Video = No |
|  | Video = Yes |
|  | NOS* |
|  | Unsure |
| Share Response | Family = No |
|  | Family = Yes |
|  | Friend = No |
|  | Friend = Yes |
|  | Mental health professional = No |
|  | Mental health professional = Yes |
|  | No one |
|  | Other |
|  | NOS* |
|  | Unsure |
| Accessibility | Access to mail |
|  | Digital literacy |
|  | Disability-related |
|  | Financial |
|  | Language |
|  | Other lack of Access |
|  | Patient grounded |
|  | Time |
|  | NOS* |
|  | Unsure |
| Privacy/Security | No concerns |
|  | Concerns |
|  | NOS* |
|  | Unsure |
| Automatic Enrollment | Opt-in |
|  | Opt-out |
|  | NOS* |
|  | Unsure |

**NOS = Not on Subject*
