# Supplementary material for: Adolescent and Young Adult Preferences for the Delivery of Caring Contacts and the Incorporation of AI: Qualitative Interview Study
Source: JMIR Form Res. 2026 Nov 11;10:e104853. doi: 10.2196/104853 (PMC13754059; doi:10.2196/104853)
Supplement: Supplementary file 4 [file formative-v10-e104853-s004.docx]

***Multimedia Appendix***

Multimedia Appendix 4. Additional Corresponding Quotations for Themes

| **Themes** | **Related Question** | **Quote** |
| --- | --- | --- |
| Perceived Usability | Preferred Delivery Method | “I do [use MyChart], but I’m not very good at navigating it. Yeah like tech is not. My strong suit.” - P2  “I would prefer a text message just because it’s the quickest way to reach me, but an email would be good too same as email or a log in through MyChart or something would suffice. But I think a text would be best.” - P4  “One thing about [patient portal] is that I don’t always receive like depending on the patient portal, I don’t always receive reminders when they reach out to me or like this person has reached out. So again it might be kind of like a mail situation where I don’t see it for a very long time.” - P5  “I would say text messaging because sometimes it can be hard to like ask your parents…like for me, my phone has a bunch of like parental controls…so I feel like texting would be the best because then you could almost always be able to use it.” - P9  “No, it’s just however. However you can, like whatever you’re doing in that moment. Just receiving something nice is nice.” - P11  “I guess, depending on the patient’s age. Their parents might like take their phone and look through everything…I would want some level of confidentiality, some level of privacy and a phone might not give that. So something through MyChart of something that’s more confidential might be better.” – P14 |
| Impersonal Nature | Response to AI | “I guess if [AI generated message] is reviewed before it’s sent I would feel comfortable with it. I guess my only concern is like it not sounding too like too generic or not like very I don’t know what the word is, like personal, I guess. But if it’s reviewed first and I guess I’d feel comfortable with it.” - P3  “Yeah, if [AI generated message] is like really generic and robotic, then no.” - P9  “And I know oftentimes the like the review process can be really fast and whatnot so like kind of taking a second to like make sure that it doesn’t sound disingenuous or AI-ey and whatnot, like putting in those more like making sure that it has kind of that personal touch to it and editing it.” - P10 |
| Transparency | Response to AI | “You can opt into this AI thing and it will be an AI and maybe put in a point about how like you’ve said it like real personal thoughts from the care team. I don’t know, something to show that it is human based. But yeah, be just so super clear. Put like. A robot emoji next to it. It’s like just be like completely face value, this is an AI because otherwise like you will do damage if someone thinks they’re going to a person and they discover it’s a robot.” - P1  “I personally would really dislike that. I’d say if it’s, if I know it’s AI, I would immediately find it like less meaningful to me. It would take away like the positive feeling I would get from it even if I do know it will be approved. I also know that like there has been in the past some disasters with AI when it comes to support for people which I know like would kind of be stopped by people overlooking it before they send it. But yeah, I personally would not personally like it to be AI.” - P5 |
